# Management challenges and outcomes for non-tuberculous mycobacterial pulmonary disease

**DOI:** 10.5588/ijtldopen.24.0140

**Published:** 2024-07-01

**Authors:** J. De Man, N. Adriaenssens, P. Van Bleyenbergh, M. Happaerts, E. André, I. Spriet, L. Dupont, N. Lorent

**Affiliations:** ^1^ Respiratory Diseases Department, and; ^2^ Department of Internal Medicine, University Hospitals Leuven, Leuven, Belgium;; ^3^ Department of Chronic Diseases, Metabolism and Aging, Laboratory of Respiratory Diseases and Thoracic Surgery (BREATHE), and; ^4^ Department of Microbiology, Immunology and Transplantation, KULeuven, Leuven, Belgium;; ^5^ Microbiology Department, and; ^6^ Department of Pharmacy, University Hospitals Leuven, Leuven, Belgium;; ^7^ Department of Pharmaceutical and Pharmacological Sciences, KULeuven, Leuven, Belgium

**Keywords:** NTM-PD, infection, guideline-based therapy, adverse events, treatment changes

Dear Editor,

Management of non-tuberculous mycobacterial pulmonary disease (NTM-PD) is challenging. In particular, identifying who will benefit most from antimicrobial therapy and determining the optimal time for treatment initiation are complex considerations for a clinician. An update of international NTM management guidelines advocates for a more aggressive approach than ‘watchful waiting’.^1^ However, treatment of NTM-PD requires a long course (≥12–18 months) of a multidrug treatment regimen, often resulting in unsatisfactory outcomes.^2^ Major contributors to suboptimal treatment outcomes are lack of effective drugs, natural or acquired resistance to antimicrobials of NTM species and frequent occurrence of drug-related adverse events (AE), which often result in poor treatment adherence, drug regimen modifications and treatment cessation.^3,4^ Data on the occurrence of AE and their impact on treatment outcomes are scarce.^5^ Therefore, we conducted a retrospective, single-centre, observational cohort study to evaluate treatment deviations, the reasons for these, and the outcomes of patients treated for NTM-PD.

We included all adults (aged ≥18 years) with NTM-PD (according to diagnostic criteria from the American Thoracic Society and the Infectious Diseases Society of America^1^) seen at the Respiratory Diseases Department of the University Hospitals Leuven, a tertiary referral centre in Belgium, from 1 January 2016 to 1 January 2021. Data on patient demographics, comorbidities, NTM history, presenting symptoms, radiological findings, microbiological results, date of NTM-PD diagnosis, treatment regimen, AE, treatment interruption/ changes and their reasons and treatment outcomes were collected through electronic patient record review. Patients were routinely followed up at 3, 6, 12, and 24 months (if applicable) and at the end of treatment. All data were censored on 1 June 2022, at loss to follow-up or death, whichever occurred first. Treatment outcome categories were based on the consensus outcome definitions proposed by the NTM Network European Trials group (NTM-NET).^6^ We performed descriptive statistics with Stata v14 (Stata Corp, College Station, TX, USA). All patients provided informed consent and ethical approval was granted by Ethics Committee Research KU/UZ Leuven, Leuven, Belgium (S62926).

We identified 63 individuals with NTM-PD. The median age at diagnosis was 63 years (IQR 42–72), with a female/male ratio of 1.5. The most common comorbidity was bronchiectasis. Three patients (5%) had fibrocavitary disease, 28 (68%) had nodular-bronchiectatic disease, and 9 (22%) had mixed pattern. The most frequently identified species were M. avium complex (MAC) in 34 patients (54%), with M. avium as the most frequent subspecies. Seventeen patients (27%) had M. abscessus complex (MAB), with M. abscessus abscessus as the most frequent subspecies. M. kansasii was present in 5 patients (8%). Forty-one patients (65%) initiated daily treatment, guideline-based for all. The drugs constituting the initial treatment regimens of M. avium complex pulmonary disease (MAC-PD) and M. abscessus complex pulmonary disease (MAB-PD) are shown in the Table. Two MAC-PD patients with macrolide sensitivity received intravenous (IV) amikacin because of cavitary disease. MAC-PD patients with macrolide resistance received IV amikacin thrice weekly (duration depending on tolerance and therapy effectiveness, mostly 2–3 months), associated with daily rifampicin, ethambutol and clofazimine. All nine patients treated for MAB-PD received an amikacin, azithromycin and tigecycline-containing regimen. Treatment-related AE occurred in 24 out of 41 (59%) cases. The most frequent toxicities were ototoxicity (n =13), gastrointestinal complaints (n =12), ocular (n = 6) and cutaneous (n = 5) toxicity. As depicted in the Table, AEs were more common in MAB-PD and led to treatment changes in respectively 30% and 90% of the MAC-PD and MAB-PD patients. Fourteen out of 41 (34%) patients discontinued treatment, either temporarily (n = 2, due to AE) or permanently (n = 12), of whom 13/14 with MAC-PD or MAB-PD, shown in the Table. The reasons for permanent treatment discontinuation were AE (n = 8), severe hospital-acquired pneumonia (HAP) and death (n = 2), and treatment failure after 46 months of treatment (n = 1). Additional treatment during follow-up was required in 3 MAC-PD patients (13%) and in 8 patients (88%) with MAB-PD because of a lack of sputum culture conversion. Among the treated patients, only 19 patients (46%) completed therapy for 12 months following sputum culture conversion. Ten patients (24%) obtained clinical and microbiological cure, 12 (29%) microbiological cure, 2 (5%) clinical cure, and 6 (15%) had treatment failure. Two patients were less than 12 months on treatment at the moment of data collection. The Table shows treatment outcomes for MAC-PD and MAB-PD. All 3 MAC-PD patients and 5 out of 8 MAB-PD patients who required additional treatment had treatment failure.

**Table.** Initial treatment, adverse events, changes and discontinuation and treatment outcomes for MAC-PD and MAB-PD.*

	MAC-PD^†^		MAB-PD^‡^	
	Macrolide-susceptible (*n* = 19) *n* (%)	Macrolide-resistant (*n* = 4) *n* (%)	Macrolide-susceptible (*n* = 5) *n* (%)	Macrolide-resistant (*n* = 4) *n* (%)
Initial treatment regimen				
AZM	19 (100)	0 (0)	5 (100)	4 (100)
RIF	19 (100)	4 (100)	0 (0)	0 (0)
EMB	18 (95)	4 (100)	0 (0)	0 (0)
CFZ	1 (5)	3 (75)	4 (80)	4 (100)
AMK	2 (11)	4 (100)	5 (100)	4 (100)
Imipenem	0 (0)	0 (0)	3 (60)	4 (100)
Tigecyclin	0 (0)	0 (0)	5 (100)	4 (100)
AEs	10 (53)	2 (50)	5 (100)	4 (100)
Regimen changes due to AE	5 (26)	2 (50)	5 (100)	3 (75)
Treatment discontinuation due to AE	7 (37)	1 (25)	2 (40)	3 (75)
Treatment completion	10 (53)	1 (25)	1 (20)	1 (25)
Treatment outcome				
Favourable outcome^§^	14 (73)	2 (50)	2 (40)	2 (50)
Treatment failure	2 (11)	1 (25)	3 (60)	2 (50)
Lost to follow-up	2 (11)	1 (25)	0 (0)	0 (0)

* All initiated treatment regimens were daily and guideline-based.

† The initial treatment regimen for MAC-PD consisted of daily AZM, EMB and RIF. If RIF or EMB were not tolerated, they were replaced by CFZ. In case of extensive or cavitary MAC-PD, intravenous AMK (as long as tolerated) ± oral CFZ was added.

‡ Initial MAB-PD treatment regimens consisted of an intensive phase with 2–3 intravenous drugs (AMK plus tigecycline and/or imipenem) in addition to oral CFZ and AZM for a duration of 2-8 weeks, depending on disease severity and tolerability. Subsequently, this changed to an oral regimen with at least two functional drugs with proven susceptibility, of which AZM and CFZ formed the backbone. AZM is considered a functional drug in macrolide-susceptible *M. abscessus* complex strains only; nevertheless, it often remains part of the regimen (although not counted as a functional drug) even in macrolide-resistant strains given the potential immunomodulatory effect in underlying bronchiectatic lung disease.

§ Favourable outcome: microbiological cure, clinical cure, or microbiological and clinical cure.

MAC-PD = *M. avium* complex pulmonary disease; MAB-PD = *M. abscessus* complex pulmonary disease; AZM = azithromycin; RIF = rifampicin; EMB = ethambutol; CFZ = clofazimine; AMK = amikacin; AE = adverse event.

This study highlights a clear unmet need: the high percentage of patients experiencing AE necessitating treatment changes while on guideline-based therapy. The main reason for modification was AE in 59% of patients. Reported occurrence rates range from 21% to 81% and are significantly higher in daily- as opposed to intermittent-dosing regimens.^7–9^ In our cohort, ototoxicity was present in 32% and was mainly related to aminoglycoside use in extensive MAC and MAB-PD, followed by gastrointestinal discomfort. Other AEs, such as hepatotoxicity, cytopenia, and cutaneous rash, tend to occur early on, unlike renal dysfunction and visual disturbance, which set in later.^10^ This underscores the importance of monitoring NTM-PD patients at regular follow-up intervals to enable appropriate action if AE occur.^11^ AE often result in treatment modifications. In our cohort, treatment required adjusting in 23 patients (56%) and discontinuing in 14 patients (34%). Given the relatively limited arsenal of effective drugs, taking into account susceptibility, treatment interactions and tolerance, modifying therapy whilst adhering to guidelines is difficult. Reported adherence to treatment guidelines is low, ranging between 9.2% and 41.9%.^12,13^ One of the reasons for the low reported adherence is likely the need for treatment modifications in inexperienced hands.^12,14^ To assist less experienced clinicians in NTM treatment decisions, experts have formed clinical advisory boards.^15^ A multidisciplinary panel of experts in NTM disease was instigated in Belgium in 2021 – and is a much-valued service.^16^

Our study has several strengths and limitations. Despite the potential selection bias and lack of generalizability of single-centre retrospective studies, the patients included in this cohort benefitted from a standardised, detailed follow-up. Treatment outcomes for NTM-PD are very much species-related. The small sample size precluded subgroup analyses and the linking of treatment modifications to outcomes.

Overall, our study shows that managing NTM-PD is challenging, with less than half of patients completing guideline-based therapy. AEs often lead to treatment modifications and discontinuation. Future research should focus on markers of disease activity to predict which patients are more likely to benefit from antimicrobial therapy, monitor treatment response, and ideally enable treatment shortening.

## References

[1] Daley CL, . Treatment of nontuberculous mycobacterial pulmonary disease: An official ATS/ERS/ESCMID/IDSA clinical practice guideline. Clin Infect Dis. 2020;71(4):E1–36. 32628747 10.1093/cid/ciaa241 PMC7768748

[2] Cowman S, . Non-tuberculous mycobacterial pulmonary disease. Eur Respi J. 2019;54(1):1900250. 10.1183/13993003.00250-2019 31221809

[3] Kwon YS, . Treatment outcomes after discontinuation of ethambutol due to adverse events in *Mycobacterium avium* complex lung disease. J Korean Med Sci. 2020;35(9):e59. 32141249 10.3346/jkms.2020.35.e59 PMC7061143

[4] Zweijpfenning S, . Treatment and outcome of non-tuberculous mycobacterial pulmonary disease in a predominantly fibro-cavitary disease cohort. Respir Med. 2017;131:220–224. 28947034 10.1016/j.rmed.2017.08.031

[5] Van Ingen J, . Management of drug toxicity in *Mycobacterium avium* complex pulmonary disease: an expert panel survey. Clin Infect Dis. 2021;73(1):E256–259. 32910814 10.1093/cid/ciaa1361 PMC8491833

[6] Van Ingen J, . Treatment outcome definitions in nontuberculous mycobacterial pulmonary disease: An NTM-NET consensus statement. Eur Respi J. 2018;51:1800170. 10.1183/13993003.00170-2018 PMC6660914 29567726

[7] Jeong BH, . Intermittent antibiotic therapy for nodular bronchiectatic *Mycobacterium avium* complex lung disease. Am J Respir Crit Care Med. 2015;191(1):96–103. 25393520 10.1164/rccm.201408-1545OC

[8] Wallace RJ, . Macrolide/azalide therapy for nodular/bronchiectatic *Mycobacterium avium* complex lung disease. Chest. 2014;146(2):276–282. 24457542 10.1378/chest.13-2538 PMC4694082

[9] Morimoto K, . Macrolide-resistant *Mycobacterium avium* complex lung disease: Analysis of 102 consecutive cases. Ann Am Thorac Soc. 2016;13(11):1904–1911. 27513168 10.1513/AnnalsATS.201604-246OC

[10] Kamii Y, . Adverse reactions associated with long-term drug administration in *Mycobacterium avium* complex lung disease. Int J. Tuberc Lung Dis 2018;22(12):1505–1510. 30606324 10.5588/ijtld.18.0171

[11] Pennington KM, . Approach to the diagnosis and treatment of non-tuberculous mycobacterial disease. J Clin Tuberc Other Mycobact Dis. 2021;24: 100244. 34036184 10.1016/j.jctube.2021.100244 PMC8135042

[12] Van Ingen J, . Poor adherence to management guidelines in nontuberculous mycobacterial pulmonary diseases. Eur Respi J. 2017;49(2): 1601855. 10.1183/13993003.01855-2016 28182571

[13] Ku JH, . Treatment of nontuberculous mycobacterial (NTM) pulmonary infection in the US bronchiectasis and NTM Registry: treatment patterns, adverse events, and adherence to American Thoracic Society/Infectious Disease Society of America Treatment Guidelines. Clin Infect Dis. 2023;76(2):338–341. 36134755 10.1093/cid/ciac788

[14] Adjemian J, . Lack of adherence to evidence-based treatment guidelines for nontuberculous mycobacterial lung disease. Ann Am Thorac Soc. 2014;11(1):9–16. 24236749 10.1513/AnnalsATS.201304-085OC PMC3972983

[15] Lemson A, . Evaluation of a national multidisciplinary meeting for non-tuberculous mycobacterial disease. IJTLD OPEN 2024;1(6):279–281. 39021452 10.5588/ijtldopen.24.0044 PMC11249658

[16] Adriaenssens N, . Addressing an unmet need in the management of nontuberculous mycobacterial infections in Belgium: 1 year experience of the NTM consilium. ERS International Congress 2023 abstracts. European Respiratory Society, 2023: p PA2176.

